# Application of binasal speculum in endoscopic endonasal surgery for lesions in sellar region

**DOI:** 10.3389/fendo.2023.1250755

**Published:** 2023-12-19

**Authors:** Xi Liu, Fan Zhang, Yibo Yin, Yankai Qiu, Xing Lv, Junchao Li, Mengyu Gao, Hong Shen, Li Liu

**Affiliations:** ^1^ Department of Neurosurgery, the First Affiliated Hospital of Harbin Medical University, Harbin, China; ^2^ Department of Anesthesiology, the First Affiliated Hospital of Harbin Medical University, Harbin, China; ^3^ Department of Cardiology, Fourth Hospital of Harbin, Harbin, China

**Keywords:** binasal speculum, binostril approach, endoscopic visualization, mucosa protection, guidance

## Abstract

**Objective:**

This study aims to access the efficacy of the binasal speculum in endoscopic endonasal surgery by evaluating clinical outcomes and examining its utility through process-based performance measures in both surgeons and assistants.

**Methods:**

A total of 59 patients with lesions in sellar region who underwent endoscopic endonasal surgery with the binasal speculum between September 2020 and March 2023 were included in this study. We assessed the extent of resection and documented postoperative nasal condition. Both surgeons and assistants completed post-use surveys to exam the utility of the binasal speculum and provide an overall grading.

**Results:**

Gross total resection (GTR) was successfully achieved in 94.9% (56/59) of patients, with subtotal resection (STR) observed in 5.1% (4/59) of patients. Intraoperative cerebrospinal fluid (CSF) leakage occurred in 23.7% (14/59) of cases, and nasoseptal flap (NSF) reconstruction was required in 55.9% (33/59) of cases. The nasal airway patency rapidly recovered within 14 days in a significant majority of patients (94.9%, 56/59). Moreover, olfactory function was regained within three months postoperatively by 91.5% (54/59) of patients. The overall post-use survey mean score was 26.4. Specifically, surgeons had a mean score of 26.5, while assistants had a slightly lower mean score of 26.2. The mean overall grading for the binasal speculum was 3. Both surgeons and assistants provided a mean overall grading of 3.

**Conclusion:**

The binasal speculum provides nasal mucosa protection and reduces the risk of an endoscopic lens clouded by mucosa or blood. It plays a crucial role in accurate guidance and facilitates the swift delivery of surgical instruments, particularly in left-blinded nasal cavities. The binasal speculum reduces the learning curve, especially for endoscopic surgeons with limited experience, while enhancing collaboration and coordination between surgeons and assistants during surgery. Both surgeons and assistants rated the overall utility of the binasal speculum as “excellent.”

## Introduction

Since 1995 the endoscopic endonasal surgery has been widely recognized as the optimal approach for transnasal pituitary tumor resection (1–7). However, a notable drawback of this technique is that blood easily blurs the lens during endoscope insertion and withdrawal in the nasal cavity. Additionally,the endoscope’s diameter of approximately 4mm occupies valuable surgical space and may restrict the maneuverability of surgical instruments.Currently, scholars have overcome these limitations through the binostril approach and continuous lens irrigation (8).

Conventional nasal retractors or speculums, are indispensable surgical instruments for the endonasal transsphenoidal microsurgical approach, in which the speculum is used to push aside the nasal septum through one nostril, rapidly creating a surgical pathway that spans 7 cm in length and 2 cm in width, all while protecting the nasal mucosa. However, the conventional nasal speculum is typically made of metal,which restricts the lateral range of endoscopic movement. Consequently, the nasal speculum is rarely used in transnasal endoscopic pituitary surgery (5). Nevertheless, owing to the significant advantage of protecting the nasal mucosa,some neurosurgeons have persistently explored the application of the conventional nasal speculum in endoscopic endonasal surgery, yielding some positive outcomes (9–13).

Linsler et al. (9) performed endoscopic transnasal transsphenoidal surgery for sellar lesions through a mononostril approach with a conventional nasal speculum.They concluded that the nasal speculum provides mucosal protection, which leads the low incidence of nasal complaints is remarkable in contrast to other results. Other researchers (11, 12) also made attempts to modify conventional nasal speculums with the aim of improving instrument maneuverability during the surgery. However, the effectiveness of these modifications has not been widely accepted.In a study by Conrad et al. (13), a comparison was made between the binostril approach (without speculum) and the mononostril approach (with speculum) in endoscopic transsphenoidal pituitary surgery. The researchers concluded that the nasal speculum had two sides to the same coin: it offered protection to the nasal mucosa, but restricted bimanual handling. They further observed that the binostril approach provided a broader working corridor, enabling the broader opening of the sphenoid sinus in craniocaudal and bilateral directions, and enhancing instrument maneuverability. As a result, the binostril approach gained widespread acceptance among the majority of researchers.

Our “binasal speculum” is a modification of the conventional nasal speculum. Unlike the conventional speculum, each blade is independently inserted into the nostril, preserving the nasal septum. It is joined using a “detachable” screw, enabling the rapid establishment of the binostril surgical pathway.In theory, the “binasal speculum” has the potential to easily establish the binostril approach in endoscopic endonasal surgery. Not only does it provide protection for the nasal mucosa, but it also creates a broader corridor for bimanual handling. The binasal speculum aims to enhance instrument maneuverability, optimize surgical efficiency, and simultaneously minimize postoperative nasal complications. In this report, we present outcomes of endoscopic endonasal surgery using the binasal speculum for 59 sellar region lesions to evaluate its effectiveness and examine its utility with process-based performance measures in surgeons and assistants.

## Method

### Patients

This retrospective study included patients who underwent endoscopic endonasal surgery by the binasal speculum for the resection of lesions in the sellar region at Harbin Medical University Affiliated First Hospital from September 2020 to March 2023. A total of 59 patients, including 29 males and 30 females, with a median age of 53 years (age range: 17–69 years), were included in the study. Patients with significant underlying medical conditions were excluded. Written informed consent was obtained from all patients prior to the surgery.

### Construct and use of the binasal speculum

**Figure 1:**
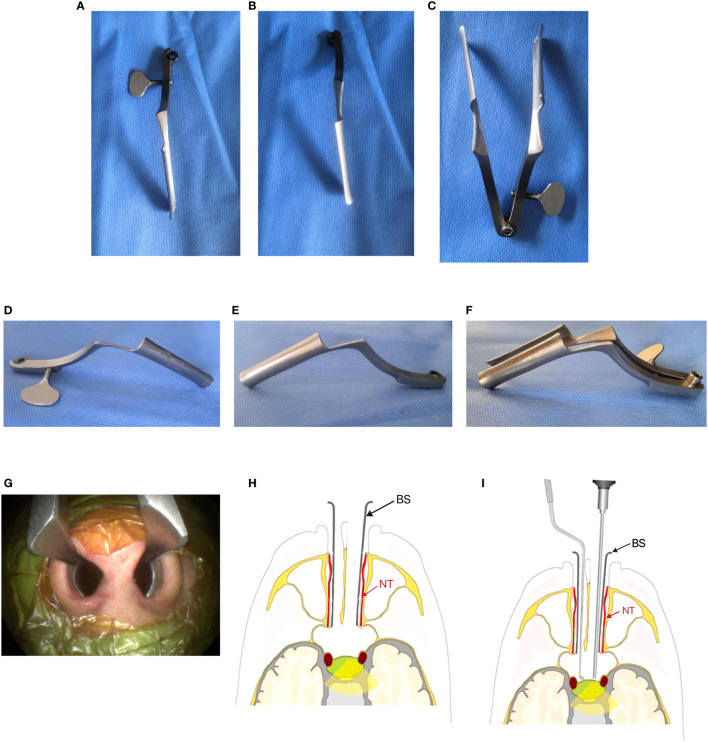
Show the design of the Binasal Speculum. **(A, D)** Superior and lateral views of the right blade. **(B, E)** Superior and lateral views of the left blade. **(C, F) **Superior view and lateral views of both blades joined by a detachable screw. **(G)** Each blade was independently inserted into each nostril. **(H, I)** Basic positioning of binasal speculum and surgical instruments in the binostril approach.(schematic drawing). NT, Nasal turbinate (red arrow); BS, blade of Binasal Speculum (black arrow).

The binasal speculum is modified from the conventional nasal speculum. The “fixed” screw of the conventional nasal speculum converted into the “detachable” screw. Each blade is independently inserted into the nostril, and then the two blades are joined using the detachable screw for easy installation. The length of each blade is 2 cm shorter from its orifice (near the screw) compared to the conventional nasal speculum. This modification makes the surgical field shallower and enhances lateral accessibility (**Figure 1**).

### Surgical procedure

**Figure 2:**
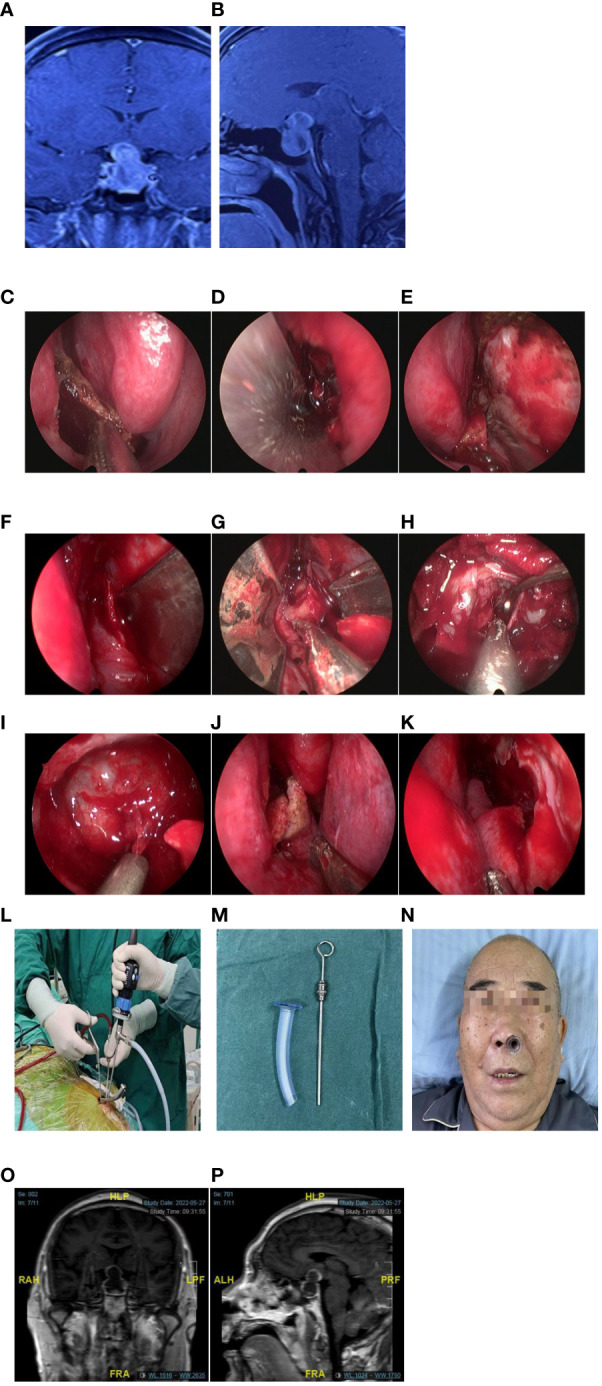
A case of giant pituitary adenoma (Knosp Grade 2) with bilateral cavernous sinus invasion and partial encasement of the internal carotid artery (ICA). [**(A, B)** T1-weighted magnetic resonance imaging (MRI) with gadolinium enhancement [**(A)** coronal section, **(B)**: sagittal section]. **(C)** Rescue Nasoseptal flap (NSF) in the right nasal cavity. **(D)** Incision made in the left nasal septal mucosa. **(E, F)** The right and left blades were separately introduced into the respective nasal cavities, aided by brain retractors. **(G)** Creation of a surgical pathway by expanding the binasal speculum. **(H)** Tumor resection. **(I)** The saddle diaphragm drop. **(J, K)** Preservation of nasal anatomy in the right and left postoperative nasalcavities.Intraoperatively, the surgeon manipulated bimanually free under the corridor, while the assistant was holding the endoscope **(L)**. **(M, N) **A shorter nasopharyngeal airway device is inserted in the left nostril to ensure postoperative ventilation and enhance patient comfort. **(O, P) **Postoperative T1-weighted MRI with gadolinium enhancement: gross total removal of the tumor [**(O)**: coronal sections, **(P)**: sagittal section].

In the nasal phase, the procedure begins by inserting the 4 mm diameter, 0°angle of view, 15 cm length endoscope (Aesculap HD, USA) into the right nasal cavity, with the right middle turbinate being laterally dislocated. Subsequently, the right sphenoid ostium and sphenoethmoidal recess are identified, and a 1.0 cm straight incision is made at the septum at the level of the proximal end of the middle turbinate, or a “rescued” nasoseptal flap (NSF) is performed as appropriate (**Figure 2C**). The right submucosal separation exposed the anterior wall of the sphenoid sinus and the right sphenoid ostium. Fracture the cartilaginous portion of the septum to the left, separating the left submucosa and exposing the left sphenoid ostium. A straight incision of about 1.0cm in length was made on the left septum mucosa, about 3.5cm away from the anterior wall of the sphenoid sinus (**Figure 2D**). Under the guidance of endoscopy, the right and left blades were introduced, respectively, with the assistance of the brain retractors. (**Figures 2E, F**) Then, the two blades were connected and joined with the detachable screw. The binasal speculum was gradually opened to the maximum extent under direct vision (**Figures 2G**).

In the sphenoidal phase, the posterior segment of the nasal septum and the anterior wall of the sphenoid sinus, along with the septations and mucosa within the sinus, are excised. Once inside the sinus, the following anatomical parts can be identified: the sella, clivus, carotid prominence, sphenoid plane, optic protuberance, and opticocarotid recess, as well as the bilateral cavernous sinuses.

**Figure 3:**
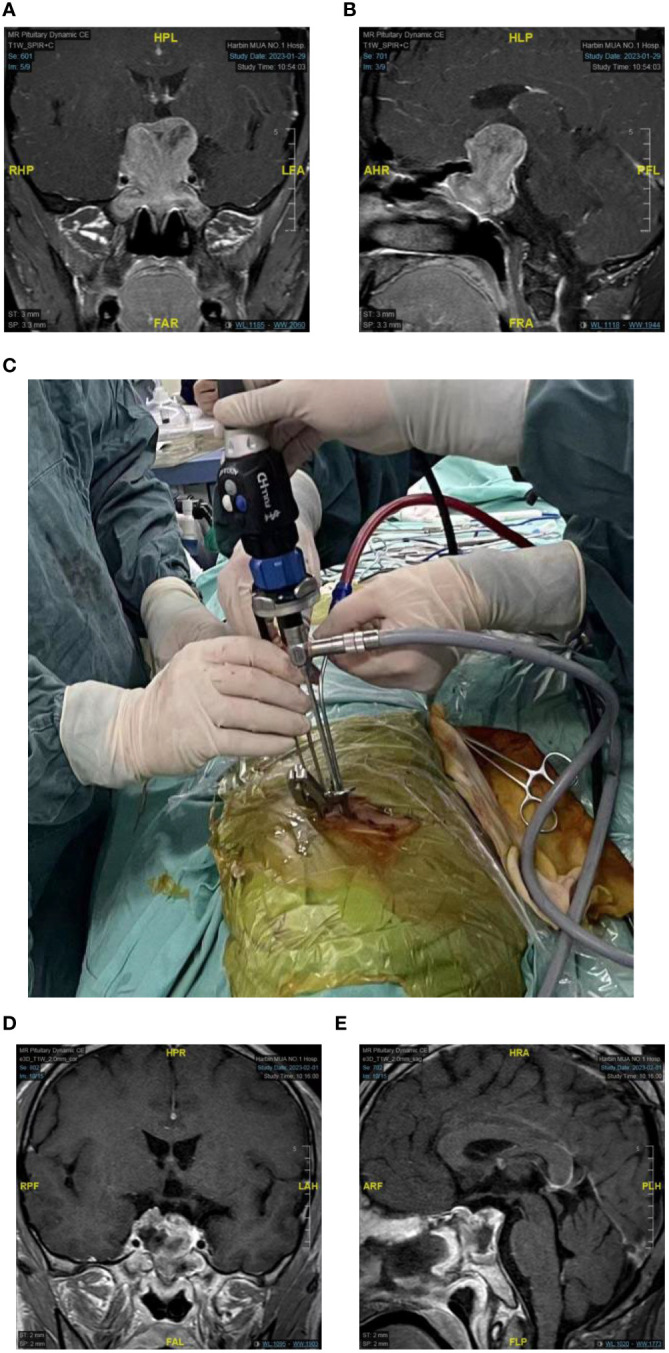
A case of a 32-year-old male presented with progressive visual decline. Preoperatively, the right eye exhibited only light perception, while the left eye had a visual acuity of 0.5, with only a small residual field of vision.The patient was diagnosed with a giant pituitary adenoma (Knosp Grade 3) with bilateral cavernous sinus invasion. **(A, B)** T1-weighted MRI with gadolinium enhancement [**(A)** coronal section, **(B)** sagittal section]. **(C)** The surgical procedure involved a four-handed, in binasitrol approach with skilled coordination in three surgeons. **(D, E)** Postoperatively, a significant and immediate improvement in visual acuity and field of vision for both eyes,MRI with gadolinium enhancement: gross total removal of the tumor [**(D)**: coronal sections, **(E)**: sagittal section].

**Figure 4:**
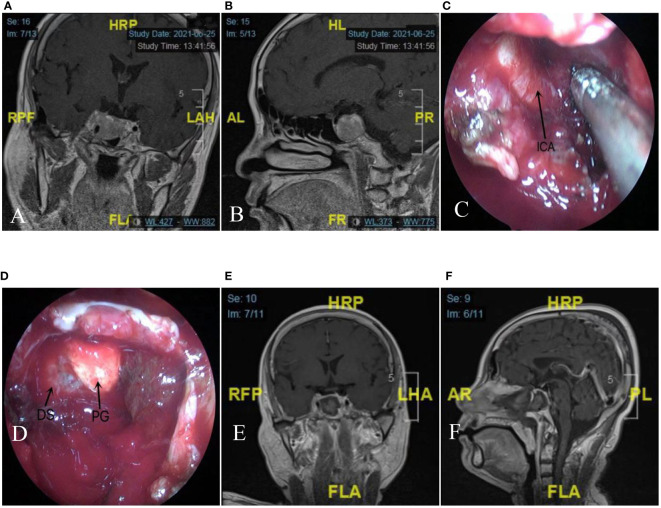
A case of giant pituitary adenoma (Knosp Grade 4) with right cavernous sinus invasion and encasement of ICA. The binasal speculum will be taken out to perform the deep lateral regions during the later stage. **(A, B)** T1-weighted MRI with gadolinium enhancement [**(A)**: coronal section, **(B)** sagittal section]. **(C)** The patient underwent eendoscopic endonasal surgery using the binasal speculum, a full exposure of ICA. **(D)** After gross total removal of the tumor, the normal anatomical structures, including the diaphragma sellae drop and the normal pituitary gland are visualized**. (E,F)** Postoperative T1-weighted MRI with gadolinium enhancement confirmed a minor residual tumor in the right cavernous sinus. [**(E)** coronal section, **(F)** sagittal section]. ICA, internal carotid artery; DS, diaphragma sellae; PG, pituitary gland(black arrow).

In the sellar phase, using bimanual technique (sometimes referred to the two-surgeons or three-surgeons, four-handed technique) (**Figures 2**–**4**), the neurosurgeon proceeded to cautious coagulation and conducted a cautious dural opening. With deliberate movements using curettes and a suction cannula, the tumor was carefully dissected from its margin and is resected piece by piece. (**Figures 2H, I**; **Supplementary Video 1**). In some cases, the binasal speculum will hinder lateral accessibility,especially when the lesion invades the cavernous sinus. Due to the binasal speculum has been used for a period of time to push aside the turbinate and mucosa, the surgical corridor of each nostril is large enough to perform bimanual procedures. The binasal speculum will be taken out to perform the deep lateral regions (**Figure 4**).

Subsequently, under direct visualization, the binasal speculum was pulled out. If no cerebrospinal fluid (CSF) leakage was detected, the sellar floor was reconstructed with artificial dura mater. Conversely, if CSF leakage occurred, the NSF was used for the sellar floor reconstruction.Both the middle turbinate and nasal mucosa were returned to their original positions at the end of the operation (**Figures 2J, K**).

Merocel sponge was packed in the right nasal cavity to prevent nasal hemorrhage while a shorter nasopharyngeal airway was placed in the left nasal cavity to ensure postoperative ventilation and enhance patient comfort (**Figure 2N**).

### Postoperative follow-up

We conducted a post-use survey, similar to Nathalia Velasquez et al. (14), to assess the utility of the binasal speculum in endoscopic endonasal surgery for both surgeons and assistants.The assessment covered nine dimensions: ease of deployment,scope and instrument movement/friction with binasal speculum,effect on movement and lateral manipulation of surgical instruments,length of blade,whether the speculum is migration during surgery,effect on manual endoscope lens cleaning,effect on lens visualization,protection of collateral structures,post-use condition.Each dimension was rated on a scale of “excellent” (3 points), “acceptable” (2 points), or “suboptimal” (1 point), with a maximum score of 27 points. The overall performance grading was also based on the same scale, with a maximum score of 3 points (**Supplementary Table 1**).

All patients underwent follow-up in the outpatient clinic or via telephone interviews. The follow-up included magnetic resonance imaging (MRI) with gadolinium enhancement to evaluate the extent of surgical resection and collect data on the recovery of nasal cavity and olfactory function. Additionally, comprehensive documentation was conducted to record any complications and nasal complaints experienced by the patients.

## Results

In this study, a total of 59 lesions in the sellar region were subjected to endoscopic endonasal surgeries using the binasal speculum to evaluate its feasibility and effectiveness. The results demonstrated a diverse range of pathologies, including 47 pituitary adenomas, 4 Rathke’s cleft cysts, 2 pituitary abscesses, 2 chordomas, 1 suprasellar arachnoid cyst, 1 metastatic lesion, 1 pituitary cyst, and 1 malignant sarcoma. Among the 47 cases of pituitary adenoma, there are 10 cases of macroadenomas (2 complicated by apoplexy, 4 recurrent pituitary adenomas), 15 cases of giant adenomas (6 pituitary adenomas with cavernous sinus invasion, 4 complicated by apoplexy, 2 recurrent pituitary adenomas), and 22 cases of pituitary microadenomas. The classification of pituitary adenomas is based on the following criteria: 1) Pituitary microadenoma: tumor diameter less than 10mm; 2) Pituitary macroadenoma: tumor diameter between 10mm and 30mm; 3) Pituitary giant adenoma: tumor diameter greater than 30mm.

Surgical resection of sellar lesions was categorized according to classification proposed by Rahimli and colleagues (15) into three types: gross total resection (GTR), subtotal resection (STR), and partial resection (PR), with proportions of (90%-100%), (70%-90%), and <70%, respectively. Among the 59 cases of endoscopic endonasal surgery using the binasal speculum, 94.9% (56/59) achieved GTR, while 5.1% (4/59) was STR. Radiosurgery is used for the small residual part in cases of STR.Notably, 53 patients received primary surgery, while 6 patients underwent revision surgery. None of the cases required conversion to microscopic surgery.

No severe postoperative complications were observed. However, intraoperative CSF leakage occurred in 23.7% (14/59) of the cases, and 55.9% (33/59) required NSF reconstruction. Two cases, one with a Rathke’s cleft cyst and the other with a sphenoid sinus arachnoid cyst, experienced postoperative CSF leakage despite the absence of intraoperative leakage. NSF reconstruction successfully resolved the postoperative CSF leakage without any additional complications on the seventh day after surgery.

During the postoperative follow-up, the majority of patients (81.3%, 48/59) achieved restoration of nasal airway patency within 3-7 days. A smaller proportion of patients (13.6%, 8/59) required 7-14 days for recovery. One patient (1.7%, 1/59) required up to 20 days for recovery, and two patients (3.3%, 2/59) experienced an extended recovery period of one month. Additionally, only a limited number of patients (6.8%, 4/59) necessitated postoperative clearance of nasal crusting due to nasal discomfort. Notably,none of the patients experienced nasal septum perforation, nasal tip deflection, or saddle nose deformity.

Regarding olfactory function, all patients experienced a temporary decrease in the immediate postoperative period. Among the patients, 28.8% (17/59) regained their olfactory senses within two weeks, while 25.4% (15/59) experienced restoration within a range of two weeks to one month. Additionally, 22.0% (13/59) of patients regained olfactory function within 1-2 months, and an additional 15.3% (9/59) required 2-3 months for recovery. Unfortunately, 8.5% (5/59) of patients had not regained their olfactory function even after three months.

Two surgeons and six assistants participated in the post-use survey, resulting in an overall mean score of 26.4. Specifically, surgeons achieved a mean score of 26.5, while assistants obtained a slightly lower mean score of 26.2. The mean overall grading for the binasal speculum was 3. Both surgeons and assistants provided an overall grading of 3, with a grading scale ranging from 1 (suboptimal) to 3 (excellent).

## Discussion

Endoscopic endonasal surgery has made significant advancements over the past two decades and has become a preferred therapeutic approach for lesions in the sellar region (1–7). The standard surgical technique is composed of three main phases: nasal, sphenoid, and sellar phases. Compared to conventional microscopic transnasal treatments, direct visualization offers superior accuracy and allows for a greater extent of lesion excision, particularly during the sellar phase. However, the 4 mm-diameter endoscope occupies a certain amount of space, which frequently requires the treatment of a large area of mucosa and the excision of one or both middle turbinates during the nasal phase. Hemostasis of the mucosa should reach the highest grade in otolaryngology, known as Boezart grade 1 (16). Extensive intervention in the nasal mucosa and excision of the middle turbinate have been documented to frequently cause postoperative nasal discomfort, requiring periodic clearance of nasal crusts every 2 to 3 weeks (3, 17).

The conventional nasal speculum is an essential instrument in microscopic transnasal surgery. Its functions include establishing a surgical pathway through the narrow nasal mucosa and minimizing the risk of mucosal injury during maneuvers with the endoscope and surgical instruments. Moreover, it improves the overall procedural efficiency. Additionally, the conventional nasal speculum exhibits a substantial hemostatic effect on mucosal bleeding. Nevertheless, its metal structure restricts the lateral movement of the endoscope and surgical instruments, leading to the exclusion of nasal speculums in the majority of current endoscopic transnasal procedures. However, studies conducted in India (11), the United States (12), Germany (13), and Japan (18) have demonstrated that the use of conventional nasal speculums in endoscopic endonasal surgery adequately protects the nasal mucosa and significantly reduces postoperative nasal morbidity.

We attempted to apply the conventional nasal speculum in endoscopic endonasal surgery, similar to previous literature findings, and discovered its advantages in protecting nasal mucosa and improving surgical efficiency. However, we also noticed that Asian individuals generally have a smaller nasal route compared to European individuals (12). The conventional single-nostril speculum is limited by the 4 mm diameter endoscope occupying a larger proportion of space, and the metal blades restricting the freedom of endoscope movement, resulting in less maneuverability compared to the binostril approach. Considering these limitations, we have developed a modified binasal speculum that retains the advantages of nasal mucosa protection, minimizes iatrogenic damage, and integrates the benefits of the binostril approach to optimize surgical maneuverability. This modified speculum is particularly suitable for the four-handed technique, ultimately leading to an overall enhancement in surgical efficiency (19).

The binasal speculum is characterized by its rapid deployment, featuring two blades that are independently inserted into each nasal cavity and joined by a detachable screw,then expanding in a manner similar to a conventional speculum. A surgical pathway can be established in just 1–4 minutes. Compared to a conventional speculum, the blade length of the binasal speculum in this study was reduced by 2 cm, resulting in a shallower surgical field and increased range for lateral maneuvers (20). Theoretically, Using the binasal speculum in binostril approach provides a broader surgical pathway compared to a conventional speculum in mononostril approach. Consequently, the binasal speculum enhances thelateral surgical field, facilitating the complete resection of tumors with cavernous sinus invasion.

The expansion of the speculum has a hemostatic effect by compressing the mucosa, and effectively protect mucosal structure.Moreover, the expansion of the speculum can dislocate the middle turbinate, thereby avoiding the need for middle turbinate excision in most patients. This promotes the recovery of the postoperative nasal mucosa and eliminates the need for periodic clearance of nasal crusting at 2- to 3-week intervals in most patients. In our research, the use of the binasal speculum significantly reduced mucosal injury, particularly in the left nasal cavity where only a straight incision of approximately 1 cm was made. Postoperatively, the right nasal cavity was packed with a merocel sponge, while a shorter nasopharyngeal airway was inserted in the left nasal cavity to provide support and ensure immediate nasal ventilation, thus enhancing patient comfort, particularly for the elderly.In the sellar phase, adequate space is created by expanding the metal speculum. In the process of lateral operation, the blades can be removed for speculum-free operation to facilitate the lateral procedure when the metal speculum obstructs the surgical instrument. In our study, surgical opening of the anterior wall of the cavernous sinus was necessary in only three cases of pituitary adenomas with cavernous sinus invasion. The speculum was taken out during the resection of the deep lateral tumor.

Pledger et al. reported that patients who underwent endoscopic endonasal surgery experienced an increase in nasal congestion symptoms during the first two weeks post-surgery, with recovery observed within four weeks (21). In contrast, in our study, the nasal congestion symptoms experienced by patients in the early postoperative period were mild. Complete resolution of nasal congestion symptoms and recovery of nasal airway patency were observed in 94.9% (56/59) of patients within two weeks after surgery. Only 4 patients (6.8%) required postoperative clearance of nasal crusting due to nasal discomfort. Previous studies have reported an incidence of anosmia ranging from approximately 10% to 14% in patients undergoing endoscopic endonasal surgery, with NSF for sellar defect reconstruction identified as a potential contributing factor (22, 23). However, our research found that only 8.5% (5/59) of patients experienced anosmia symptoms with three patients who underwent NSF for sellar defect reconstruction, and one patient who received successful intraoperative repair of CSF leakage from the cribriform plate. These findings demonstrate a decrease compared to the reported incidences in the previous literature. Thus, the theoretical and practical results of this study provide evidence of the beneficial protective effect of the binasal speculum on nasal function. Binasal speculum can protect mucosal tissue, and our results are consistent with the findings in the published literature (10, 14).

Currently, the majority of nasal speculum used worldwide in endoscopic surgery are single-nostril speculum. Scholars from India (11), the United States (12), Germany (13), and Japan (18) have continuously explored the use of the single-nostril speculum. However, the limitation of restricting the maneuverability of surgical instruments remains unavoidable and Conrad et al. (13) has demonstrated that the mononostril approach restricts the range of instrument movement compared to the binostril approach.Only two reports regarding the use of the binasal speculum in the binostril approach can be found in the literature (10, 14). Our binasal speculum design shares similarities with Shin M.’s low-profile nasal speculum (Fujita Medical Instruments) (10), but it features a simpler design with only one detachable screw, in contrast to his two screws. Moreover, while Shin M.’s speculum is primarily intended for the submucosa approach, our binasal speculum offers greater flexibility in its application. In some cases, we were able to remove the speculum quickly and perform speculum-free operations. Furthermore, Velasquez et al. (14) reported that the Nasal Access Guide (NAG) (SPIWay, LLC, California, USA), made of carbon fiber, is relatively soft and consists of independent barrel-like structures. As a result,it lacks the ability to dislocate nasal turbinates, often resulting in the need for one or both turbinates removal during the procedure. Additionally, it obstructs the surgical field in the direction of the nasal septum. In contrast, the binasal speculum, made of metal, enables the dislocation of nasal turbinates while minimizing endoscope contamination caused by nasal mucosal blood stains. Moreover, the two blades of the binasal speculum are deployed only on the outer sides of the nasal cavity, ensuring an unobstructed view in the direction of the nasal septum. In this study, the utility of the binasal speculum was consistently evaluated favorably by surgeons and assistants in a post-use survey, with a mean score of 26.4, frequently rating its overall utility as “excellent” (mean score: 3). This evaluation is better than the findings reported by Velasquez et al., where the mean score in their post-use survey of the NAG was 23.33 out of 27, and the overall grading was 2.75 out of 3. We consider that these findings may be attributed to the dislocation of nasal turbinates and the unobstructed view of the nasal septum.

Endoscopic endonasal surgery is associated with a steep learning curve, which hinders the promotion of endoscopic neurosurgery. This study introduces the modified binasal speculum as a tool to accelerate the acquisition of endonasal endoscopic techniques among neurosurgeons. It aims to reduce the learning curve and facilitate the widespread adoption of endoscopic neurosurgery, bridging the gap between novice and expert surgeons. This speculum is particularly suitable for surgeons with limited endoscopy experience. It plays a crucial guiding role and greatly facilitates the rapid passage of the endoscope and instruments through the nasal cavity, increasing the efficiency of surgery, particularly in the blinded nasal cavity (left side for right-handed surgeons). Traditionally, surgeons heavily rely on sensory perception to navigate the nasal cavity while requiring swift delivery of surgical instruments such as scissors, bipolar forceps, and dissectors in their dominant hand.In Velasquez’s report (14), surgical instruments and powered tools were passed an average of 17 and 18 times, respectively, during a brief 10-minute observation period. This indicates that there are potentially hundreds of instrument passes throughout the entire surgical procedure. Consequently, the surgical pathway established by the speculum allows surgical instruments to easily access the surgical site, thereby minimizing the risk of collateral instrument-related tissue damage from the blind passage and boosting the surgeon’s confidence, reducing the occurrence of unforeseen complicationss (24, 25).

Endoscopic endonasal surgery requires the collaboration of two surgeons.Precise collaboration between the surgeon and assistants is crucial in endoscopic endonasal surgery. A proficient surgeon relies heavily on an equally experienced assistant to get the best surgical outcomes. The binasal speculum can mitigate the difficulties in collaboration and coordination between the surgeon and assistants, particularly during the initial stages of their partnership. Moreover, it reduces the prerequisites for the assistant’s proficiency in endonasal endoscopic techniques, enabling even those with limited experience to participate proficiently in the complex surgical procedures.

Furthermore, the binasal speculum improves the surgical fluency of both the surgeon and assistants, particularly in the four-handed, binasitrol approach. This improvement in overall efficiency ultimately leads to high-quality surgical outcomes (24–26).

Additionally, we used the binasal speculum in surgeries involving optic nerve decompression and repair of CSF rhinorrhea. The binasal speculum also played a significant role in these procedures.

This study presents several potential limitations. Firstly, the assessment of surgical efficiency and fluency did not consider operation time, which varied significantly based on different sellar pathologies and the collaboration proficiency between the surgeon and assistants. The impact of these variations on operation time is significant. Secondly, the study lacked comparative analyses before and after the introduction of the binasal speculum. Thirdly, the study was the retrospective research by the single-center, there were 59 patients in the research, which made the statistical analysis more difficult. There were only 8 neurosurgeons employing this instrument in the endoscopic endonasal surgery in the study. The multi-institutional studies are necessary to investigate the efficiency and feasibility of the binasal speculum in the future. In the next research, we will enroll more and more patients and neurosurgeons with diverse geographical locations and varying surgical experience levels, the statistical analysis was employed to demonstrate the efficiency and feasibility of the binasal speculum.

## Conclusion

The binasal speculum effectively protects the nasal mucosa and reduces the risk of an endoscopic lens clouded by mucosa or blood. Additionally, it facilitates nasal ventilation and olfactory recovery, resulting in shorter recovery times. Moreover, the binasal speculum plays a crucial role in accurate guiding and facilitating swift delivery of surgical instruments, particularly in the left-blinded nasal cavities. This feature is especially beneficial for limited experienced endoscopic surgeons, as it simplifies the complexity of the procedure and reduces the learning curve.

Furthermore, it contributes to improving collaboration and coordination between the surgeon and the assistant during surgery. Consequently, the binasal speculum improves the efficiency and fluency of surgery. Both surgeons and assistants rated the overall utility of the binasal speculum as “excellent.”

## Data availability statement

The raw data supporting the conclusions of this article will be made available by the authors, without undue reservation.

## Author contributions

XiL, FZ and LL conceived and designed the study. YY, YQ and XingL organized the database. XiL and FZ performed statistical analysis. XiL, FZ and LL wrote the first draft of the manuscript. MG, HS and LL wrote portions of the manuscript. All authors contributed to article and approved the submitted version.
