# Supplementary material for: Application of binasal speculum in endoscopic endonasal surgery for lesions in sellar region
Source: Front Endocrinol (Lausanne). 2023 Dec 19;14:1250755. doi: 10.3389/fendo.2023.1250755 (PMC10763232; doi:10.3389/fendo.2023.1250755)
Supplement: Supplementary file 1 [file Table_1.docx]

Supplementary Table 1

Post-use survey of binasal speculum completed by surgeons and assistants

|  | 3. | 2 | 1 |  |
| --- | --- | --- | --- | --- |
| Attribute | “Excellent” | “Acceptable” | “Suboptimal” |  |
| Ease of deployment | Easily folded,  deployed, and unfurled. | Folded, deployed, and unfurled with moderate effort. | Cannot fold, deploy, or unfurl |  |
| Scope and instrument  movement/friction with binasal speculum | Negligible friction between binasal speculum and surgical tools (i.e., endoscope and instruments) during maneuvers. | Moderate friction and interference between binasal speculum and surgical tools (i.e., endoscope and instruments) during  maneuvers. | Friction inhibits use of surgical tools (i.e., endoscope and instruments) with binasal speculum and can even render them unusable |  |
| Effect on movement and lateral manipulation of surgical instruments. | Binasal speculum requires negligible additional force to stretch with nares, does not hinder tool maneuvers. | Binasal speculum requires moderate force to stretch with nares slightly hindering tool maneuvers. | Binasal speculum does not stretch with nares, completely hindering desired tool maneuvers. |  |
| Length of blade | Length of binasal speculum was the right length to bypass structures  and provide a working corridor without obstructing view. | Length of binasal speculum was okay. The length to bypass structures and provide a working corridor without obstructing view could be improved. | Length of binasal speculum must be  improved. It did not allow me to effectively bypass structures and provide a working corridor with  out obstructing view. |  |
| Whether the speculum is migration during surgery | Binasal speculum remains stable during surgical maneuvers. | Binasal speculum migrates moderately during high-load maneuvers  with surgical tools. | Binasal speculum migrates significantly during all maneuvers with surgical tools,even lead to its dislodgement |  |
| Effect on manual  endoscope lens  cleaning | Binasal speculum significantly reduced  need to withdraw scope for manual cleaning. | Binasal speculum somewhat helped to  reduce need to withdraw scope for manual cleaning. | Binasal speculum made no difference in or increased need to withdraw scope for manual cleaning. |  |
| Effect on lens visualization | Binasal speculum significantly reduced  frequency of lens smudging obscuring adequate visualization | Binasal speculum somewhat reduced frequency of lens smudging obscuring adequate visualization. | Binasal speculum made no difference in or increased frequency of lens smudging obscuring adequate visualization. |  |
| Protection of collateral  structures | Binasal speculum signifificantly helped  to reduce trauma to  surrounding mucosa and  structures. | Binasal speculum somewhat helped to  reduce trauma to surrounding mucosa and  structures. | Binasal speculum made no difference in or increased trauma to surrounding mucosa and  structures. |  |
| Post use condition | No damage or deformation | Moderate damage or deformation | Complete damage or deformation |  |
| Overall performance | Binasal speculum was easy to use and  provided significant utility, protect the nasal mucosa and reduce the contamination of the endoscopic lens improved performance during  maneuvers. | Binasal speculum was somewhat easy  to use and provided some utility, somewhat improved performance during maneuvers. | Binasal speculum was difficult to use  and/ or did not provide any utility, more trouble to use than not to use. |  |

Note: surgeons and assistants were instructed to circle or mark the box indicative of their assessment. Cumulative scores were tabulated, for a possible point total of 27 for the survey and 3 for the overall assessment.
